# Aberrant HPO Axis Alterations and Autoimmune Abnormalities in PCOS Patients with DOR: A Retrospective Analysis

**DOI:** 10.3390/jcm12165212

**Published:** 2023-08-10

**Authors:** Xueying Geng, Zhihong He, Zhouzhou Bao, Wen Di, Zhuowei Gu

**Affiliations:** 1 Department of Obstetrics and Gynecology, Renji Hospital, School of Medicine, Shanghai Jiaotong University, Shanghai 200127, China; 2 Center for Reproductive Medicine, Renji Hospital, School of Medicine, Shanghai Jiaotong University, Shanghai 200135, China; 3 Shanghai Key Laboratory for Assisted Reproduction and Reproductive Genetics, Shanghai 200135, China; 4 Shanghai Key Laboratory of Gynecologic Oncology, Renji Hospital, School of Medicine, Shanghai Jiaotong University, Shanghai 200127, China; 5 State Key Laboratory of Oncogenes and Related Genes, Shanghai Cancer Institute, Renji Hospital, School of Medicine, Shanghai Jiaotong University, Shanghai 200127, China

**Keywords:** PCOS, DOR, autoimmune antibodies, HPO axis, glucolipid metabolism

## Abstract

Background: There is a group of polycystic ovary syndrome (PCOS) patients in clinic who have diminished ovarian reserve (DOR) in combination. This study was designed to evaluate the differences in glucolipid metabolism, hypothalamic–pituitary–ovarian (HPO) axis-related parameters, and autoimmune antibodies in PCOS patients with and without DOR. Methods: A total of 2307 PCOS patients, including 1757 patients with PCOS alone and 550 patients who have both PCOS and DOR, were enrolled in this retrospective study. Parameters of glucolipid metabolism, HPO axis-related parameters, and autoimmune antibodies were measured and analyzed. Results: The prevalence of DOR among all patients with PCOS was 23.84%. Many HPO axis-related parameters, such as follicle-stimulating hormone (FSH), luteinizing hormone (LH), estradiol (E2), and prolactin (PRL) were significantly different in PCOS with DOR compared with PCOS without DOR. The FSH levels were positively correlated with LH, testosterone (T), and androstenedione (AD) levels, but had no association with glucolipid metabolism after adjusting for body mass index (BMI). Moreover, anti-ovarian antibody (AOAb) and anti-21-OH antibody (21-OHAb) levels were significantly elevated in PCOS patients with DOR. Conclusions: PCOS patients with DOR showed more chaotic HPO axis hormone levels and elevated autoimmune antibodies, suggesting that autoimmune factors may be the cause of DOR in women with PCOS.

## 1. Introduction

Polycystic ovary syndrome (PCOS) is the most commonly occurring reproductive and metabolic disorder, affecting 6–20% of women of reproductive age [1]. PCOS is characterized by the presence of hyperandrogenism, oligo/anovulation, and polycystic ovarian morphology [2]. Women’s metabolic, reproductive, cardiovascular, and psychological health can all be significantly impacted by PCOS [3]. PCOS is the result of interactions of multiple genetic factors, environmental factors, and epigenetic influences. However, the pathogenesis of PCOS has been unclear.

It is well known that the abnormal hypothalamic–pituitary–ovarian (HPO) axis is related to PCOS [4]. In PCOS, there are several kinds of dysfunctions of the HPO axis, such as abnormal gonadotropin-releasing hormone (GnRH) pulse frequency, elevated levels of luteinizing hormone (LH), and an imbalance in the ratio of LH/follicle-stimulating hormone (FSH), adrenal, and ovarian androgen excess [5]. Mounting evidence suggests that patients with PCOS have an increased body mass index (BMI), higher prevalence of insulin resistance and impaired glucose metabolism, and more unfavorable lipid profiles than controls [6,7,8].

Diminished ovarian reserve (DOR) is described as a challenging condition of poor quantity and quality of ovarian follicles and premature decline in fertility. The concept of DOR is heterogeneous, and the definition of DOR is still not clear. It is uncomplicated to distinguish DOR from poor ovarian responders (POR) and premature ovarian failure (POF), the latter two having been clearly identified [9]. According to the Bologna criteria and others [10,11,12,13,14], the diagnosis criteria are based on a pre-cycle assessment of antral follicle count (AFC), FSH, and Anti-Müllerian hormone (AMH). According to reports, women with DOR comprise about 24% of the infertile population and 32% of the in vitro fertilization (IVF) cycles [15,16]. Furthermore, the prevalence of DOR has risen from 19% to 26% over the last decade [14]. In addition to genetic factors, age, enzyme deficiencies, environmental factors, unhealthy lifestyle, and iatrogenic causes influences on DOR, autoimmune factors have also been reported to influence DOR [17].

It is generally accepted that PCOS and DOR are representative of two extremes of ovarian reserve [18,19]. A recent study performed by our group has characterized the different metabolic characteristics between PCOS patients with DOR and without DOR [12]. BMI and homeostasis model assessment insulin resistance (HOMA-IR) are significantly higher in the simple PCOS group than in the PCOS with DOR group. However, the rationale for better glucose and lipid metabolism in patients with PCOS along with DOR has not been found. 

This study is one of the first attempts to use a large number of samples to describe the prevalence of DOR in women with PCOS, and to evaluate the correlation between different manifestations. We assessed HPO axis-related parameters, autoimmune antibodies, as well as glucose and lipid metabolic parameters that may identify patients with PCOS-DOR. We found that abnormal HPO axis or autoimmune factors may be the possible pathogenic reason why women with PCOS have DOR.

## 2. Materials and Methods

### 2.1. Patients

This was a retrospective study including a total of 2307 PCOS patients, 1757 PCOS patients without DOR and 550 PCOS patients with DOR, who applied to Shanghai Ren Ji Hospital’s obstetrics and gynecology outpatient clinic from June 2015 to February 2022. Written informed consent was obtained from all patients for the study, approved by Ren Ji Hospital Ethics Committee (KY2020-047). The study was conducted single-centered.

### 2.2. Patient Selection and Clinical Measurements

The diagnosis of PCOS was made according to the diagnostic criteria recommended by the Rotterdam criteria in 2003 [20], following at least any two out of all three features: oligo- and/or anovulation, clinical and/or biochemical signs of hyperandrogenism, and polycystic ovaries. Patients were excluded if they had unexplained infertility, congenital abnormalities, recurrent abortion, congenital adrenal hyperplasia, Cushing syndrome, or androgen-secreting tumors. 

Evaluation of the study participants began with a history and physiological examination. Patients’ age, pregnancy, abortion and birth counts, gynecological history, presence of systemic disease (hypertension, diabetes, chronic liver and kidney disease, autoimmune diseases, etc.), previous operations history, family history, smoking, alcohol, and drug use were in the questionnaire (Table S1). We classified phenotypes of PCOS patients according to the Rotterdam consensus as follows: (A) oligo/anovulation (O), hyperandrogenism (HA), and polycystic ovaries (PCO); (B) O and HA; (C) HA and PCO; and (D) O and PCO.

Oligo/anovulation was defined by the presence of oligo-amenorrhea (fewer than 8 per year or absence of no bleeding for 3 months or more, excluding pregnancy). Hyperandrogenemia was diagnosed by evaluating testosterone (T), androstenedione (AD), sex hormone-binding globulin (SHBG), and the free androgen index (FAI). The FAI was calculated in T (nmol/L)/[SHBG (nmol/L)] × 100. The presence of hirsutism, acne, or alopecia was taken as the clinical appearance of hyperandrogenism. In the ultrasonography, the PCO image was evaluated with the presence of 12 or more follicles in each ovary measuring 2–9 mm in diameter, and/or increased ovarian volume (>10 mL) [17]. 

Body mass index (BMI) was calculated from the height and weight measured at each participant’s research visit. Waist–hip ratio (WHR) was calculated from the waist and hip circumferences.

Transvaginal ultrasonography was routinely conducted. AFC was defined as the number of bilateral follicles (2–10 mm in diameter) in early follicular phase.

The diagnosis of PCOS with DOR was made in the presence of PCOS complicated by a basal FSH concentration of >10 to <25 IU/L, an FSH/LH ratio of >3, or a basal E2 concentration of >80 pg/mL [11,12,14].

### 2.3. Biochemical Measurements

In the early follicular phase (days 2–5) of the menstrual cycle, 5 mL of blood samples were taken and tested in the clinical laboratory of Ren Ji Hospital. LH, FSH, E2, prolactin (PRL), T, AD, SHBG, and thyroid-stimulating hormone (TSH) levels were measured using an IMMULITE 2000 immunoassay system (Diagnostic Products Corporation, Siemens Healthineers, Erlangen, Germany). Triglycerides (TG), total cholesterol (TC), low-density lipoprotein (LDL), high-density lipoprotein (HDL), fasting blood glucose (GLU), and fasting insulin (FIN) levels were measured using a Roche Cobas 8000-c702 analyzer (Roche Diagnostics, Basel, Switzerland). The following formula was used to determine insulin resistance (HOMA-IR): fasting plasma insulin (mIU/L) × fasting plasma glucose (mmol/L)/22.5 [21].

In order to study autoimmune antibodies and kisspeptin, 2 mL venous blood samples were collected in anticoagulant-free biochemistry tubes, centrifuged at 2500 rpm for 25 min, and stored at −80 °C until the day of analysis. Human serum was detected using the Human Anti-Ovarian Antibody ELISA kit (MyBioSource, San Diego, CA, USA), Human Anti-21-OH Antibody ELISA kit (MyBioSource, San Diego, CA, USA), and KiSS-1 (112–121) Amide/Kisspeptin-10/Metastin (45–54) Amide (Human) EIA Kit (Phoenix Pharmaceuticals, Mannheim, Germany), respectively. All procedures were carried out using the standard protocols.

### 2.4. Statistical Analysis

All statistical analyses were performed with IBM SPSS Statistics 28.0.0.0. Descriptive statistics are presented with percentage, frequency, mean, and standard deviation (SD) while evaluating the findings obtained in the study. Pearson’s chi-square test was used to compare the differences in frequencies between groups. Student’s *t*-test was used in the analysis to compare differences between PCOS patients with and without DOR. In order to analyze the relationship between FSH and other variables, we stratified the FSH data into four groups according to the upper (FSH = 8.78 IU/L) and lower limits (FSH = 3.85 IU/L) of the normal detection range (FSH < 3.85, 3.85 ≤ FSH < 8.78, 8.78 ≤ FSH ≤ 10, FSH > 10, IU/L). One-way ANOVA was used to compare differences among the four PCOS patient groups according to FSH level. Pearson correlation was used to assess the strength of correlation among variables. To assess the association between FSH and each endocrine and metabolic parameter, multiple linear regression analysis was conducted with various endocrine and metabolic indicators as dependent variables. FSH (A) and BMI (B) were used as independent variables (Y = β0 + β1*A + β2*B). *p* < 0.05 were considered significant.

## 3. Results

### 3.1. Characteristics of Study Participants

Characteristics of a total of 2307 PCOS patients, 1757 PCOS patients without DOR and 550 PCOS patients with DOR, are provided in Table 1. PCOS patients with and without DOR were similar in regard to age. PCOS patients with DOR had lower BMI and waist–hip ratio (WHR) compared to survivors without PCOS (mean BMI 24.23 vs. 22.77, mean WHR 0.83 vs. 0.81, respectively). HPO axis-related parameters were significantly different between the two groups. LH, E2, and PRL levels were significantly higher in PCOS with DOR compared with PCOS without DOR; among them, LH levels were discrete, but still had statistical significance (10.76 vs. 15.66 IU/L, 163.60 vs. 460.38 pmol/L, and 12.92 vs. 14.29 μg/L, respectively). Incredibly, FSH levels dropped slightly in PCOS patients with DOR (6.62 vs. 6.58 IU/L). It seems that the LH/FSH ratio was elevated in PCOS patients with DOR (1.63 vs. 2.32), while the FSH/LH ratio remained unchanged (0.88 vs. 1.00). Androgen (T and AD) levels did not differ between the two groups. Due to the increase in SHBG levels, FAI decreased significantly in PCOS with DOR (10.29 vs. 7.66, respectively). AMH levels were much lower in PCOS with DOR (8.22 vs. 7.27, respectively), as well as antral follicle count (AFC, 36.05 vs. 29.98, respectively). In PCOS patients with DOR, the counts of pregnancies and abortions did not differ compared to PCOS patients without DOR, but there was a downward trend in the occurrence of births (0.19 vs. 0.15, respectively). Consistent with the BMI results, the majority of glucose and lipid metabolism parameters were significantly improved in PCOS patients with DOR than in PCOS patients without DOR, including fasting blood glucose, fasting insulin, HOMA-IR, TG, and HDL-C. 

### 3.2. Prevalence of DOR in Women with PCOS

A paper published in 2017 showed that the prevalence of DOR in Chinese PCOS patients was 16.9% [11], then a former study performed by our group calculated the prevalence of DOR among patients with PCOS from 2015 to 2019 and found the prevalence of DOR among all patients with PCOS was 20.8% [12]. At present, we recounted the prevalence of DOR in patients with PCOS from 2015 to 2022, and found that the prevalence had further increased. Among a total of 2307 PCOS patients included in the study, 550 patients suffered from both PCOS and DOR, so at the time of this study, the prevalence of DOR among all patients with PCOS was 23.84%. 

The most common form of PCOS subtypes in both groups was HA + O + PCO (hyperandrogenism + oligo/anovulation + polycystic ovary) (Table 2), and there was a significant decline in the proportion of patients with three phenotypes (HA + O + PCO) at the same time in PCOS patients with DOR relative to PCOS patients without DOR (73.82% vs. 70.55%, respectively). Among the remaining three subtypes, no clear tendency was shown.

### 3.3. Clinical, Endocrine and Metabolic Characteristics in Patients with PCOS According to FSH Levels

In the ovaries, endogenous FSH results in a clear stimulation of preantral follicle growth and FSH stimulates follicle growth moderately in synergy with other stimulating factors such as androgens and growth differentiation factor-9 (GDF9) [22]. Levels of serum FSH are of great significance for reflecting pituitary endocrine function and the functional state of the ovary. Basal FSH has been used to estimate ovarian reserve for more than 30 years [23]. In the former study, we have found abnormal elevated FSH levels in some PCOS patients. In order to analyze the relationship between FSH and other characters, we stratified all PCOS patients into four groups according to the upper (FSH = 8.78 IU/L) and lower limits (FSH = 3.85 IU/L) of the FSH detection range (FSH < 3.85, 3.85 ≤ FSH < 8.78, 8.78 ≤ FSH ≤ 10, FSH > 10, IU/L). In this way, we discovered that the FSH levels were positively correlated with LH, T, and AD levels. In the population with FSH > 3.85 IU/L, patients’ FSH levels were inversely associated with BMI, fasting blood insulin (FINS), and HOMA-IR, but showed a positive tendency with TC (Table 3).

### 3.4. Associations of Basal FSH Levels with Endocrine and Metabolic Variables in PCOS Patients

Table 4 **(Middle column)** shows the associations between basic FSH (bFSH) levels and other variables in all 2307 PCOS patients, displaying a significantly negative association between FSH levels and BMI, WHR, E2, PRL, FINS, and HOMA-IR levels. Moreover, we found that FSH levels were positively associated with LH, T, and SHBG levels. Table 4 **(Right column)** shows the associations between bFSH levels and other variables in 550 PCOS patients with DOR, exhibiting a strong positive connection between FSH levels and LH levels. There were negative associations between FSH levels and age, BMI, WHR, E2, FINS, and HOMA-IR levels. Taken together, we figured out that FSH levels were synchronized with LH levels.

### 3.5. The Independent Influence of BMI in Correlations between FSH and Metabolic Variables 

After adjusting for BMI, multiple linear regression analysis revealed that FSH showed no association with lipid metabolic factors and was not directly connected with WHR and HOMA-IR levels (Table 5). However, it is interesting that FSH exhibited a substantial correlation with LH, E2, PRL, and T, which indicated a strong linkage-type disruption of HPO axis hormones

The outcome was the same when we narrowed our attention to PCOS patients with DOR after controlling for BMI. Correlations between FSH and metabolic variables vanished, whereas correlations between FSH and LH, E2, and T were revealed (Table S2).

### 3.6. Autoimmune Abnormalities and Kisspeptin Levels in PCOS Patients with DOR 

**Figure 1:**
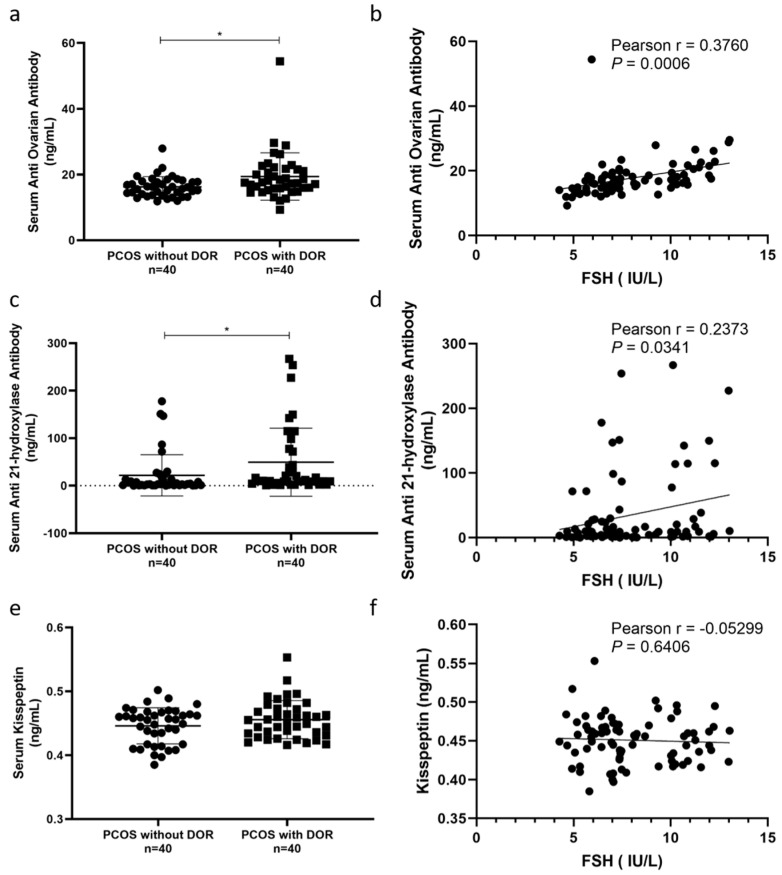
Levels of AOAb and 21−OHAb were increased in PCOS patients with DOR compared to PCOS patients without DOR, and kisspeptin levels showed no differences. (**a**,**c**,**e**) ELISA was used to detect AOAb, 21−OHAb, and kisspeptin in the serum of PCOS patients with DOR (n = 40) and PCOS patients without DOR (n = 40), respectively. Data are presented as the mean ± SD. * *p* < 0.05 (Student’s *t*-test). (**b**,**d**,**f**) Correlations between AOAb, 21−OHAb, and kisspeptin and FSH were calculated separately using the Pearson correlation analysis. Pearson correlation coefficient (r) was used to determine the strength of the correlation between the two.

In order to explore whether autoimmune factors and HPO axis-related factors are different between PCOS patients with DOR and without DOR, we selected indicators such as anti-ovarian antibody (AOAb), anti-21-OH antibody (21-OHAb), and kisspeptin, and tested them in a cohort of PCOS patients (Table S3). We found that AOAb and 21-OHAb were significantly elevated in the serum of PCOS patients with DOR (Figure 1a,c), and kisspeptin also tended to increase, although without statistical difference (Figure 1e). At the same time, we found that the level of FSH was proportional to the level of AOAb (Figure 1b), and proportional to the level of 21-OHAb (Figure 1d), but there was no significant correlation with the level of kisspeptin (Figure 1f). 

## 4. Discussion

We found a linear relationship between basal FSH levels and HPO axis hormones in this retrospective analysis of PCOS patients with and without DOR (Table 3 and Table 4). Even after potential confounders were taken into account, this connection persisted (Table 5). Additionally, we discovered an improved metabolic condition in PCOS patients with DOR compared to PCOS patients without DOR, which was mainly attributable to the variety of patients’ BMI through analysis (Table 1). Our study is one of the first to investigate associations between the marker of ovarian reserve FSH and endocrine and metabolic characteristics in PCOS patients. The findings revealed that PCOS patients with DOR had more aberrant HPO axis alterations. At the same time, for the first time, we have clearly confirmed that there were more autoimmune abnormalities in PCOS patients with DOR, and the levels of autoimmune antibodies including 21-OHAb and AOAb are increased (Figure 1).

In recent years, clinicians in China have described a phenomenon that PCOS patients with high bFSH or FSH/LH ratio or high basal estradiol should be diagnosed as DOR. Meanwhile, they found that the prevalence of DOR in PCOS patients was 16.9% [11], and then a former study performed by our group showed the prevalence of DOR among patients with PCOS from 2015 to 2019 was 20.8% [12]. In this study, we recounted the prevalence of DOR in patients with PCOS from 2015 to 2022, and found that the prevalence had further increased to 23.84%. A former study found that primary ovarian insufficiency occurred more among women with PCOS compared with women without PCOS (3.73% vs. 0.44%; *p* < 0.001), suggesting that prior PCOS was an independent risk factor for development of reduced ovarian reserve function [24]. PCOS patients with DOR face a reduced number of primordial follicles and a low response to gonadotropins treatment. Compared with patients with PCOS alone, the treatment is more complicated, and the process of ovulation induction faces more risks and adverse outcomes. Therefore, this type of group is worthy of more attention.

It is generally accepted that PCOS and DOR are representative of two extremes of ovarian reserve. Therefore, it seems contradictory that a patient has both PCOS and DOR. Exploring the similarities and differences between PCOS and DOR helps to understand the potential mechanism of this syndrome. From an etiological perspective, there appear to be some commonalities between PCOS and DOR. 

Autoimmune factors are the important intersection between PCOS and DOR. The human ovary is commonly the target of an autoimmune attack leading to ovarian dysfunction [25]. The autoimmune etiology of DOR included the presence of lymphocytic oophoritis, autoantibodies to ovarian antigens, and association with other autoimmune disorders [26]. Many associations between PCOS and autoimmune disorders have been reported [27,28,29,30]. The ovarian dysfunction and progressive loss of reserve function could be caused by autoimmune oophoritis in mice. In humans, this autoimmune disease is characterized by serum ovarian autoantibodies, such as anti-ovarian antibody (AOAb), anti-21-OH antibody (21-OHAb) [31]. It seems that AOAs can bind to steroid hormone-producing cells, causing steroid cell antibodies (StCAs) [31,32]. The main target antigen is 21-OH [33]. A study showed that positive anti-ovarian antibodies for at least one isotype were present in 15 (44%) of 34 of the PCOS women [34]. Our research pleasantly found that AOAb and 21-OHAb were significantly elevated in the serum of PCOS patients with DOR. In particular, AOAb has a significant positive correlation with FSH (Figure 1). The above results suggest that women with PCOS-DOR should be alert to the possible existence of autoimmune oophoritis. Autoimmune thyroid diseases are also associated with ovarian reserve. Idiopathic ovarian reserve insufficiency was accompanied by lower serum levels of AMH, which was associated with more frequent anti-TPO positivity rather than thyroid function or anti-TG positivity. Moreover, AMH levels were significantly lower in PCOS with Hashimoto’s thyroiditis group [28,35,36]. Unfortunately, the heterogeneity of the syndrome and the variety of antigens lead to indeterminate results. In subsequent experiments, we should also extend exploration to studies of thyroid antibodies. From what has been discussed above, our results confirmed more severe autoimmune abnormalities in PCOS patients with DOR. The detection of autoimmune antibodies in PCOS patients with DOR can be valuable. 

Many genetic factors play a role in PCOS or DOR pathogenesis. Genome-wide association studies identified several PCOS candidate loci: DENND1A, INSR, YAP1, C9orf3, RAB5B, HMGA2, TOX3, SUMO1P1/ZNF217, THADA, FSHR, and LHCGR from Han Chinese populations [37,38], and FSHR/LHCGR, DENND1A, RAB5B, and THADA from European populations [39]. PCOS is related to androgen exposures during the prenatal period, epigenetic factors, diets, lifestyles, endocrine disruptors, and emotional disorders [40,41]. Many DOR cases attributed to an idiopathic cause have a genetic component; a systematic review showed genes associated with DOR, FMR1, IGF1, IGF2, IGF1R, IGF2R, GDF9, FSHR, ESR1, AMH, LHCGR, and GREM1 [42]. We can figure out that candidate genes in PCOS and candidate genes in DOR have overlaps, and these overlaps can be verified in PCOS patients with DOR in the future. Given the homogeneity of POI and DOR, observations have identified that novel gene bionetworks that regulate primordial follicle assembly were linked to POI and PCOS, suggesting that abnormalities in the ovarian primordial follicle might be a component of POI and PCOS later in life as some of the genes involved were in common [43]. FSH receptor polymorphisms found in women with PCOS have been reported to be associated with higher serum FSH levels and POR to gonadotropins [44,45]. Genetic polymorphisms in GnRH and gonadotrophic hormone receptors affect the phenotype of PCOS; FSHR Ser (680) was related to higher levels of FSH and a higher frequency of hyperandrogenism [45]. This is consistent with our finding of high FSH in PCOS and inspires us to further validate whether the FSHR polymorphism is also present in our cohorts.

In addition, from an epigenetic transgenerational inheritance perspective, studies found ancestral homology induced by environmental toxicant between polycystic ovary syndrome and primary ovarian insufficiency. After ancestral vinclozolin or DDT exposures, transgenerational epigenetic changes in F3 generation rats appear to contribute to ovary dysregulations and disease susceptibilities that can occur in later life [46]. Neonatal exposure to endocrine disruption (estradiol E2 or hexestrol DES) was found to affect ovarian stem cells and their differentiation (neo-oogenesis) and primordial follicle assembly in adult ovaries. Neonatal exposure to E2 leads to features typical of polycystic ovary syndrome, whereas DES-treated ovaries show POI-like manifestations [47].

A recent research hotspot, glycolysis, may also be situated at the intersection of the pathogenesis of PCOS and POI. On the one hand, PCOS is characterized by disturbed follicular development, increased primordial follicle activation, and failure of antral follicle growth. PCOS patients usually exhibit disturbed glycolysis and pyruvate metabolism in follicular fluid, as well as a decreased proportion of primordial follicles, and the enhanced glycolysis may lead to the recruitment of primordial follicle pools, which may result in an increase in the density of small preantral follicles. On the other hand, down-regulated glycolysis-related genes lncRNA-ALDOA and HK3 are present in granulosa cells of patients with POI. Thus, decreased glycolytic capacity may lead to POI through a decrease in the number of growing follicles [48].

Serum FSH has been accepted as a classic and reliable marker for ovarian reserve. Our study found that after adjusting BMI, the significant correlation between FSH and metabolic indicators disappeared, indicating that BMI could be used as an independent factor to affect the metabolic level of PCOS patients with DOR and PCOS patients without DOR. In Table 1, we found that the mean values of GLU and FINS were within the normal range for PCOS patients with and without DOR, as well as the mean values of lipid metabolism-related parameters. This may be due to the fact that we did not use unsupervised hierarchical cluster analysis to differentiate patients according to abnormalities in glucose and lipid metabolism. According to the new classification of PCOS, the “metabolic” group, characterized by higher BMI, glucose, and insulin levels, and lower SHBG and LH levels, accounts for approximately 37–39% of the total amount of cases of PCOS [49]. Even within this subtype, the mean values of glucose metabolism indicators in PCOS patients are within the normal range. In fact, impaired glucose tolerance in PCOS patients is mainly manifested by elevated 2 h postprandial blood glucose and 2 h postprandial insulin. In the paper, we found that GLU, INS, HOMA-IR, and TG were better in those with PCOS with DOR than in those with PCOS without DOR (Table 1). Moreover, as FSH increased, BMI decreased significantly, along with a significant decrease in FINS and HOMA-IR (Table 3). Besides reproductive and endocrine responses, FSH, along with LH/hCG, also plays a key role in the regulation of ovarian metabolism, particularly glucose metabolism [50,51]. Although FSH and LH/hCG can increase glucose uptake and utilization by preovulatory granulosa cells [51], FSH is more potent in promoting glucose uptake and storage in preovulatory granulosa cells, whereas LH/hCG has a stronger glycolytic activity [51]. FSH induces the expression of IRS-2, which activates PI3K, Akt, and glucose uptake [50], which combines reproductive processes with energy homeostasis. FSH-stimulated glucose uptake and glycogen levels are defective in GCs from both insulin-resistant and non-insulin-resistant PCOS patients [51]. In the former section, we mentioned that hyper-glycolysis is prevalent in granulosa cells of PCOS patients, and high FSH might alleviate this hyperactivity and normalize metabolism in granulosa cells. Subsequently, we will continue to explore whether there is an improvement in glucose metabolism in granulosa cells of PCOS patients with DOR and the specific effects of this improved metabolic condition on follicular development and fertility in PCOS patients with DOR.

When we delved into the causes of the differences in BMI between the two groups, we thought that androgens might be one of the root causes. The free androgen index of PCOS without DOR group was significantly higher than that of PCOS with DOR group (Table 1). Androgen excess and insulin resistance in PCOS are related to each other, and both are related to abnormal lipid metabolism. The close relationship between the three factors affects the occurrence and development of PCOS [8,52,53]. Therefore, exploring the etiology of androgen changes in women with PCOS with DOR is also a topic worthy of further study.

The results of Table 3 suggest that in PCOS patients with DOR there is a phenomenon of simultaneous elevation of FSH and LH, and with the increase of FSH, the level of E2 presents a “U”-shaped curve, while the level of T keeps rising. It is well known that both the absolute level of circulating LH and LH/FSH levels are significantly elevated in PCOS women compared to controls. This is due to increased amplitude and frequency of LH pulses [20]. A recent study has shown that approximately 30% to 50% of patients with polycystic ovary syndrome have high serum basal LH levels and nearly 5% of women with high LH polycystic ovary syndrome have poor ovarian response [54]. In DOR patients, due to the decrease in the number of follicles, the insufficient secretion of estrogen and progesterone, and the weakening of negative feedback, the pituitary gland will secrete more FSH, resulting in an increase of the FSH levels. Some studies believe that the mechanism of acupuncture in DOR treatment could be activating the dopamine system via regulating the HPO axis [55]. Hypothalamic control of reproduction is coordinated by the pulsatile release of gonadotropin-releasing hormone (GnRH) from the arcuate nucleus of the hypothalamus through the pituitary portal system, which acts on the pituitary gland and regulates the secretion of luteinizing hormone (LH) and follicle-stimulating hormone (FSH). The latter acts on the ovaries to stimulate follicular growth and promote the release of sex steroids, such as testosterone (T), estradiol (E2), and progesterone (P4) [5,56]. On the one hand, such hormonal changes might be hypothalamic-pituitary abnormalities or negative feedback abnormalities; on the other hand, PCOS patients with DOR might have follicle stagnation in the preantral follicular phase, with antral follicles reduced accordingly. Feedback from developing ovarian antral follicles which secrete activins, inhibins, and follistatins in addition to sex steroids weakened [23], resulting in higher FSH values. Overlaying these results with the pathophysiology of PCOS, PCOS patients with DOR are more inclined to the reproductive subtype rather than the metabolic subtype according to the novel PCOS classification [57]. Therefore, we investigated the kisspeptin levels in the serum of PCOS patients, which is the main factor controlling gonadotrophins secretion. During the periovulatory period, GnRH neurons directly transmit a kisspeptin-controlling signal to the pituitary, which is a pulsatile secretion of GnRH, thus stimulating LH and FSH pulsatile release [58]. We found that kisspeptin showed no difference between PCOS patients with DOR and without DOR (Figure 1). In the future, we need to improve and examine more HPO axis-related indicators to clarify the cause of gonadotropin elevation. Patients with PCOS combined with DOR face more complex clinical dilemmas than normal PCOS patients. It is recommended to add AMH and antral follicle count (AFC) examinations for PCOS patients with DOR who have fertility requirements, to check their follicle status, in order to contribute to subsequent treatment.

Several limitations must be considered in the interpretation of our work. First, we focused our attention on a large sample of PCOS patients and compared them in two groups based on whether they had DOR comorbidities. Because the patients came from outpatient clinics, there was a lack of a control group in the data, but this did not affect the accuracy of our results. Our cohort was comprised of women from China. This may limit generalizability to women in other regions so it will be meaningful to assess the prevalence of DOR in women with PCOS from the rest of the world in the future. Additionally, while we demonstrated an association between basal FSH levels and LH, T, and E2 concentration in PCOS patients with DOR, and found an independent role of metabolism in the disease, we have not delved into the mechanics so far. In the future, we will further improve the data from patients, including AMH, AFC, autoimmune antibodies, and hypothalamic-pituitary-related parameters such as kisspeptin. Furthermore, we will continue to follow up with the patients and pay more attention to their ovulation induction and pregnancy outcomes, to have a more comprehensive understanding of the prognosis of PCOS with DOR.

## 5. Conclusions

In this article, we updated the prevalence of DOR in Chinese PCOS patients using large sample data, and the results showed an increased trend. Compared with PCOS patients without DOR, PCOS patients with DOR showed more chaotic HPO axis hormone levels, but better glucose and lipid metabolism. There was a substantial positive linear connection between FSH and LH in PCOS patients with DOR, suggesting an abnormality of the HPO axis. BMI was an independent factor in the association between FSH and metabolic variables. Low BMI may be a protective factor in disease severity in PCOS patients with DOR. Autoimmune antibodies elevated in PCOS patients with DOR, suggesting that autoimmune factors may be the cause of DOR in some women with PCOS.

## Figures and Tables

**Table 1:** Clinical and biochemical data from PCOS patients with and without DOR.

	PCOS without DOR (n = 1757)	PCOS with DOR (n = 550)	*p*-Value	Reference Value
Age, years	25.95 ± 5.16	25.93 ± 5.14	0.907	N/A
BMI, kg/m^2^	24.23 ± 4.89	22.77 ± 4.67	<0.0001	18.5–23.9 Overweight: 24–27.9 Obese: ≥28
Waist/hip ratio	0.83 ± 0.06	0.81 ± 0.07	<0.0001	0.67–0.8
LH, IU/L	10.76 ± 6.84	15.66 ± 16.94	<0.0001	2.12–10.89
FSH, IU/L	6.62 ± 1.48	6.58 ± 3.09	0.686	3.85–8.78
E2, pmol/L	163.60 ± 61.29	460.38 ± 344.52	<0.0001	55.7–469.2
Prolactin, μg/L	12.92 ± 6.72	14.29 ± 7.64	<0.0001	3.34–26.72
T, nmol/L	2.28 ± 0.89	2.33 ± 0.85	0.256	0.35–2.6
AD, ng/mL	3.50 ± 1.28	3.63 ± 1.49	0.727	0.3–3.3
SHBG, nmol/L	34.38 ± 22.91	45.18 ± 27.60	<0.0001	18.2–135.5
FAI	10.29 ± 9.55	7.66 ± 6.58	<0.0001	N/A
LH/FSH	1.63 ± 0.99	2.32 ± 1.67	<0.0001	N/A
FSH/LH	0.88 ± 0.57	1.00 ± 1.58	0.090	N/A
AMH, ng/mL	8.22 ± 4.09	7.27 ± 3.56	0.030	2–6.8
AFC †	36.05 ± 13.24 (n = 919)	29.98 ± 10.11 (n = 255)	<0.0001	
GLU, mmol/L	5.14 ± 0.72	5.05 ± 0.69	0.018	3.9–6.1
FINS, mIU/L	10.20 ± 10.20	8.24 ± 6.94	<0.0001	1.9–23
HOMA-IR	2.42 ± 2.84	1.91 ± 1.88	<0.0001	N/A
TSH, mIU/L	2.39 ± 2.06	2.24 ± 1.43	0.086	0.27–4.20
TC, mmol/L	4.67 ± 1.30	4.66 ± 0.83	0.873	<5.72
TG, mmol/L	1.18 ± 0.77	1.01 ± 0.59	<0.0001	<1.7
HDL-C, mmol/L	1.36 ± 0.29	1.43 ± 0.29	0.015	0.9–2.00
LDL-C, mmol/L	2.74 ± 0.77	2.66 ± 0.74	0.059	<3.4
Pregnancy	0.37 ± 0.80	0.34 ± 0.79	0.417	
Birth	0.19 ± 0.46	0.15 ± 0.40	0.091	
abortion	0.18 ± 0.53	0.19 ± 0.55	0.839	

Data are presented as mean ± SD. The two-tailed Student’s *t*-test was used to compare differences between PCOS patients with and without DOR. *p* < 0.05 means significant difference between means. PCOS, polycystic ovary syndrome; DOR, diminished ovarian reserve; BMI, body mass index; LH, luteinizing hormone; FSH, follicle-stimulating hormone; E2, estradiol; T, testosterone; AD, androstenedione; SHBG, sex hormone-binding globulin; FAI, free androgen index; AMH, Anti-Müllerian hormone; AFC, antral follicle count in both ovaries; GLU, fasting blood glucose; FINS, fasting blood insulin; HOMA-IR, homeostasis model assessment-insulin resistance; TSH, thyroid-stimulating hormone; TC, total cholesterol; TG, triglyceride; HDL-C, high-density lipoprotein cholesterol; LDL-C, low-density lipoprotein cholesterol. † The number of patients who were included in each analysis is provided if it differs from the total number of the group.

**Table 2:** Proportion of various subtypes in PCOS patients with or without DOR (%).

	PCOS without DOR (n = 1387)		PCOS with DOR (n = 455)		*p*-Value
Subtypes	Counts	Rate (%)	Counts	Rate (%)	
HA + O + PCO	1024	73.82	321	70.55	<0.01
HA + O	66	4.76	27	5.93	0.320
HA + PCO	70	5.05	27	5.93	0.462
O + PCO	227	16.37	80	17.59	0.546

Pearson’s chi-square test was performed to compare the differences in frequencies between groups. *p* < 0.05 means significant difference. PCOS, polycystic ovary syndrome; DOR, diminished ovarian reserve; HA, hyperandrogenism; O, oligo/anovulation; PCO, polycystic ovary.

**Table 3:** Clinical and biochemical data from PCOS according to the normal detection range of FSH levels.

	FSH < 3.85 (n = 161)	FSH 3.85–8.78 (n = 1874)	FSH 8.78–10 (n = 166)	FSH > 10 (n = 106)	*p*-Value
Age, years	26.08 ± 5.44	25.89 ± 5.19	26.35 ± 4.81	26.03 ± 4.62	0.707
BMI, kg/m^2^	24.12 ± 4.41 ^a^	24.03 ± 4.89 ^a^	23.26 ± 4.98 ^ab^	21.77 ± 4.63 ^b^	<0.0001
Waist/hip ratio	0.82 ± 0.06 ^ab^	0.83 ± 0.07 ^a^	0.81 ± 0.06 ^ab^	0.80 ± 0.07 ^b^	0.0005
LH, IU/L	5.28 ± 3.37 ^a^	11.24 ± 7.48 ^a^	15.91 ± 10.86 ^b^	27.88 ± 28.65 ^c^	<0.0001
FSH, IU/L	2.66 ± 0.77 ^a^	6.44 ± 1.18 ^b^	9.33 ± 0.35 ^c^	11.34 ± 1.41 ^c^	<0.0001
E2, pmol/L	566.96 ± 436.92 ^a^	204.72 ± 146.68 ^b^	202.99 ± 152.61 ^b^	349.52 ± 385.13 ^c^	<0.0001
Prolactin, μg/L	17.03 ± 8.35 ^a^	12.95 ± 6.75 ^bc^	12.00 ± 5.71 ^b^	14.75 ± 8.30 ^ac^	<0.0001
T, nmol/L	2.05 ± 0.73 ^a^	2.30 ± 0.90 ^b^	2.41 ± 0.90 ^b^	2.40 ± 0.75 ^b^	0.0008
AD, ng/mL	3.34 ± 1.25	3.52 ± 1.33	3.62 ± 1.40	3.87 ± 1.51	0.957
SHBG, nmol/L	42.84 ± 32.56 ^ab^	35.51 ± 23.34 ^b^	41.01 ± 24.84 ^ab^	47.61 ± 26.83 ^a^	<0.0001
FAI	7.90 ± 7.55 ^a^	10.03 ± 9.35 ^b^	8.40 ± 6.94 ^ab^	7.58 ± 6.96 ^a^	0.0003
LH/FSH	2.02 ± 1.28 ^ac^	1.75 ± 1.14 ^b^	1.70 ± 1.16 ^bc^	2.36 ± 2.16 ^ad^	0.002
FSH/LH	0.96 ± 1.60	0.92 ± 0.87	0.84 ± 0.56	0.78 ± 0.68	0.280
AMH	7.74 ± 4.63	7.98 ± 4.04	8.69 ± 5.08	7.45 ± 4.38	0.789
GLU, mmol/L	5.06 ± 0.58	5.13 ± 0.73	5.08 ± 0.76	5.04 ± 0.59	0.226
FINS, mIU/L	9.82 ± 7.91 ^a^	10.05 ± 9.35 ^a^	7.96 ± 5.33 ^ab^	6.79 ± 4.99 ^b^	<0.0001
HOMA-IR	2.26 ± 2.03 ^a^	2.38 ± 2.82 ^ab^	1.88 ± 1.70 ^bd^	1.55 ± 1.26 ^cd^	<0.0001
TSH, mIU/L	2.46 ± 1.97	2.35 ± 2.00	2.29 ± 1.34	2.46 ± 1.30	0.981
TC, mmol/L	4.58 ± 0.84	4.65 ± 1.27	4.78 ± 0.86	4.80 ± 0.94	0.330
TG, mmol/L	1.03 ± 0.56	1.16 ± 0.74	1.11 ± 0.91	1.06 ± 0.63	0.127
HDL-C, mmol/L	1.39 ± 0.30	1.36 ± 0.29	1.40 ± 0.33	1.46 ± 0.31	0.278
LDL-C, mmol/L	2.69 ± 0.77	2.71 ± 0.77	2.83 ± 0.74	2.70 ± 0.78	0.295

Data are presented as mean ± SD. One-way ANOVA was performed to compare differences among the four PCOS patient groups according to FSH level. If significantly different, then multiple comparison analysis method was used. Different letters (^a,b,c,d^) indicate significant differences, while the same letter indicates no significant difference. BMI, body mass index; LH, luteinizing hormone; FSH, follicle-stimulating hormone; E2, estradiol; T, testosterone; AD, androstenedione; SHBG, sex hormone-binding globulin; FAI, free androgen index; AMH, Anti-Müllerian hormone; GLU, fasting blood glucose; FINS, fasting blood insulin; HOMA-IR, homeostasis model assessment-insulin resistance; TSH, thyroid-stimulating hormone; TC, total cholesterol; TG, triglyceride; HDL-C, high-density lipoprotein cholesterol; LDL-C, low-density lipoprotein cholesterol.

**Table 4:** Associations between bFSH and other variables in all PCOS patients and in PCOS with DOR.

	r	
	all PCOS (n = 2307)	PCOS with DOR (n = 550)
Age, years	0.271	−0.021
BMI, kg/m^2^	−0.102 ****	−0.154 ***
Waist/hip ratio	−0.085 *	−0.145 *
LH, IU/L	0.438 ****	0.521 ****
E2, pmol/L	−0.159 ****	−0.251 ****
Prolactin, μg/L	−0.091 ****	−0.084
T, nmol/L	0.096 ****	0.140 **
AD, ng/mL	0.018	0.108 *
SHBG, nmol/L	0.008 **	0.094
FAI	−0.046	−0.008
AMH	0.07634	0.08875
GLU, mmol/L	−0.019	0.006
FINS, mIU/L	−0.095 ****	−0.127 **
HOMA-IR	−0.080 ***	−0.108 *
TC, mmol/L	0.030	0.083
TG, mmol/L	0.036	0.028
HDL-C, mmol/L	0.031	0.050
LDL-C, mmol/L	0.007	−0.002

Pearson correlation was used to assess the strength of correlation among variables. * *p* < 0.05, ** *p* < 0.01, *** *p* < 0.001, **** *p* < 0.0001. PCOS, polycystic ovary syndrome; DOR, diminished ovarian reserve; BMI, body mass index; LH, luteinizing hormone; FSH, follicle-stimulating hormone; E2, estradiol; T, testosterone; AD, androstenedione; SHBG, sex hormone-binding globulin; FAI, free androgen index; AMH, Anti-Müllerian hormone; GLU, fasting blood glucose; FINS, fasting blood insulin; HOMA-IR, homeostasis model assessment-insulin resistance; TC, total cholesterol; TG, triglyceride; HDL-C, high-density lipoprotein cholesterol; LDL-C, low-density lipoprotein cholesterol.

**Table 5:** Multiple linear regression among FSH, BMI, and metabolic parameters in PCOS patients.

Parameters	β1	β2	Multiple R	R Squared	Adjusted R Squared
	FSH	BMI			
Waist/hip ratio	−0.0004	0.008 ****	0.578	0.334	0.332
LH, IU/L	2.211 ****	−0.227 ****	0.447	0.200	0.200
E2, pmol/L	−19.080 ****	−4.303 ****	0.189	0.036	0.035
Prolactin, μg/L	−0.319 ****	-0.046	0.093	0.009	0.008
T, nmol/L	0.0442 ****	0.009 *	0.106	0.011	0.010
AD, ng/mL	0.076	−0.002	0.016	0.0003	−0.001
SHBG, nmol/L	0.082	−2.329 ****	0.470	0.221	0.220
FAI	−0.007	0.665 ****	0.367	0.134	0.133
GLU, mmol/L	0.003	0.042 ****	0.281	0.079	0.078
FINS, mIU/L	−0.202 *	0.998 ****	0.519	0.269	0.268
HOMA-IR	−0.041	0.261 ****	0.484	0.235	0.240
TC, mmol/L	0.025	0.026 ****	0.109	0.012	0.011
TG, mmol/L	0.001	0.057 ****	0.379	0.144	0.143
HDL-C, mmol/L	−0.002	−0.027 ****	0.452	0.204	0.201
LDL-C, mmol/L	0.014	0.0456 ****	0.290	0.084	0.083
TSH, mIU/L	0.0120	0.0445	0.034	0.001	0.0002

Multiple linear regression analysis was used with various endocrine and metabolic indicators as dependent variables, and FSH (A) and BMI (B) as independent variables (Y = β0 + β1*A + β2*B). * *p* < 0.05, **** *p* < 0.0001. FSH, follicle-stimulating hormone; BMI, body mass index; LH, luteinizing hormone; E2, estradiol; T, testosterone; AD, androstenedione; SHBG, sex hormone-binding globulin; FAI, free androgen index; GLU, fasting blood glucose; FINS, fasting blood insulin; HOMA-IR, homeostasis model assessment-insulin resistance; TC, total cholesterol; TG, triglyceride; HDL-C, high-density lipoprotein cholesterol; LDL-C, low-density lipoprotein cholesterol; TSH, thyroid-stimulating hormone.

## Data Availability

The data presented in this study are available on request from the corresponding author. The data are not publicly available due to privacy restrictions.

## Supplementary Materials

The following supporting information can be downloaded at: https://www.mdpi.com/article/10.3390/jcm12165212/s1, Table S1. Characteristics of systemic diseases and family history of patients in Table1. Table S2. Multiple linear regression among FSH, BMI and metabolic parameters in PCOS patients with DOR. Table S3. Clinical and biochemical data from PCOS patients with and without DOR for ELISA.

## Abbreviations

PCOS	polycystic ovary syndrome
DOR	diminished ovarian reserve
HPO	hypothalamic–pituitary–ovarian
LH	luteinizing hormone
FSH	follicle-stimulating hormone
AMH	Anti-Müllerian hormone
AFC	antral follicle count
GnRH	gonadotropin-releasing hormone
BMI	body mass index
HOMA-IR	homeostasis model assessment insulin resistance
T	testosterone
AD	androstenedione
SHBG	sex hormone-binding globulin
FAI	free androgen index
WHR	waist–hip ratio
PRL	prolactin
TSH	thyroid-stimulating hormone
TG	triglycerides
TC	total cholesterol
LDL	low-density lipoprotein
HDL	high-density lipoprotein
GLU	fasting blood glucose
FIN	fasting insulin
AOAb	anti-ovarian antibody
21-OHAb	anti-21-OH antibody
