# Supplementary material for: Aberrant HPO Axis Alterations and Autoimmune Abnormalities in PCOS Patients with DOR: A Retrospective Analysis
Source: J Clin Med. 2023 Aug 10;12(16):5212. doi: 10.3390/jcm12165212 (PMC10455465; doi:10.3390/jcm12165212)
Supplement: Supplementary file 1 [file jcm-12-05212-s001.zip › jcm-2500075-supplementary.pdf]

Supplementary Material

Supplementary Tables

Table S1. Characteristics of systemic diseases and family history of patients in Table1

	PCOS without DOR(n=1757)	PCOS with DOR (n=550)
Systemic diseases — no. (%)		
Diabetes	43 (2.45)	13 (2.36)
Hypertension	11 (0.63)	3 (0.55)
Chronic liver disease	70 (3.98)	4 (0.73)
Chronic kidney disease	1 (0.06)	1 (0.18)
Autoimmune diseases	116 (6.60)	41 (7.45)
Family history of Diabetes— no. (%)		
No	1170 (66.59)	390 (70.91)
First-degree relatives	262 (14.91)	68 (12.36)
Second-degree relatives	316 (17.99)	92 (16.73)
First and second-degree relatives	9 (0.51)	0 (0)

Table S2. Multiple linear regression among FSH, BMI and metabolic parameters in PCOS patients with DOR

Parameters	β1	β2	Multiple R	R squared	Adjusted R squared
	FSH	BMI			
Waist/hip ratio	-0.001	0.009****	0.596	0.355	0.350
LH, IU/L	2.759****	-0.215	0.519	0.270	0.267
E2, pmol/L	-29.200****	-3.322	0.258	0.066	0.063
Prolactin, μg/L	-0.158	0.185*	0.137	0.019	0.015
T, nmol/L	0.039**	0.004	0.139	0.019	0.016
AD, ng/mL	0.040	-0.014	0.104	0.011	0.006
SHBG, nmol/L	0.080	-3.014****	0.509	0.259	0.256
FAI	0.172	0.646****	0.447	0.200	0.196
GLU, mmol/L	0.011	0.044****	0.289	0.083	0.080
FINS, mIU/L	-0.053	1.021****	0.686	0.471	0.469
HOMA-IR	-0.008	0.261****	0.638	0.407	0.405

**TC**, mmol/L	0.0268*	0.024**	0.156	0.024	0.020
**TG**, mmol/L	0.018*	0.059****	0.455	0.207	0.204
HDL-C, mmol/L	-0.003	-0.03181****	0.519	0.269	0.257
LDL-C, mmol/L	0.010	0.04892****	0.305	0.093	0.089
TSH, mIU/L	-0.0001	0.04197**	0.134	0.018	0.014

Multiple linear regression analysis was performed with various endocrine and metabolic indicators as dependent variables, FSH (A) and BMI (B) as independent variables ($Y = \beta 0 + \beta 1 * A + \beta 2 * B$). *p < 0.05, **p < 0.01, ***p < 0.001, ****p < 0.0001.

Table S3. Clinical and biochemical data from PCOS patients with and without DOR for ELISA.

	PCOS without DOR(n=40)	PCOS with DOR(n=40)	P-value
Age, years	23.95 ± 5.07	25.35 ± 5.55	0.177
BMI, kg/m2	23.56 ± 4.41	24.60 ± 6.61	0.421
Waist/hip ratio	0.82 ± 0.06	0.81 ± 0.07	0.425
LH, IU/L	12.74 ± 10.09	20.89 ± 6.62	<0.0001
FSH, IU/L	6.73 ± 1.25	9.34 ± 2.45	<0.0001
E2, pmol/L	169.36 ± 48.07	203.75 ± 229.39	0.362
Prolactin, µg/L	11.64 ± 4.34	14.30 ± 6.94	0.043
T, nmol/L	3.02 ± 0.51	2.26 ± 0.82	<0.0001
AD, ng/mL	5.09 ± 0.74	3.65 ± 1.67	<0.0001
SHBG, nmol/L	30.52 ± 21.03	40.94 ± 28.80	0.071
FAI	14.69 ± 9.11	8.65 ± 6.97	0.001
LH/FSH	3.14 ± 1.00	1.28 ± 0.94	<0.0001
FSH/LH	0.35 ± 0.09	1.70 ± 1.61	<0.0001
GLU, mmol/L	5.07 ± 0.44	5.09 ± 0.67	0.860
FINS, mIU/L	10.19 ± 8.60	8.95 ± 8.11	0.506
HOMA-IR	2.38 ± 2.20	2.03 ± 2.07	0.477
TSH, mIU/L	2.28 ± 1.18	2.48 ± 1.81	0.582
TC, mmol/L	4.68 ± 0.62	4.68 ± 0.86	0.985
TG, mmol/L	1.03 ± 0.61	1.15 ± 0.72	0.428
HDL-C, mmol/L	1.44 ± 0.30	1.40 ± 0.30	0.643
LDL-C, mmol/L	2.79 ± 0.65	2.76 ± 0.77	0.831

Data are presented as mean ± SD. Student t-test was used in the analysis of the data (age) with normal distribution. Mann-Whitney test was used to compare differences between PCOS patients with and without DOR in non-normal distribution parameters.
